# Long-term prognostic significance of rising PSA levels following radiotherapy for localized prostate cancer – focus on overall survival

**DOI:** 10.1186/s13014-017-0837-5

**Published:** 2017-06-14

**Authors:** Carla Freiberger, Vanessa Berneking, Thomas-Alexander Vögeli, Ruth Kirschner-Hermanns, Michael J. Eble, Michael Pinkawa

**Affiliations:** 1 Department of Radiation Oncology, RWTH Aachen University, Pauwelsstrasse 30, 52072 Aachen, Germany; 2 Department of Urology, RWTH Aachen University, Pauwelsstrasse 30, 52072 Aachen, Germany; 3 Department of Urology/Neuro-Urology, Friedrich-Wilhelms-University Bonn, Sigmund-Freud-Str. 25, 53105 Bonn, Germany; 4 Department of Radiation Oncology, MediClin Robert Janker Klinik, Villenstr. 8, 53129 Bonn, Germany

**Keywords:** Prostate cancer, Radiotherapy, Brachytherapy, Prostate specific antigen, Biochemical failure, Overall survival

## Abstract

**Background:**

The aim of this study was to evaluate the long-term prognostic significance of rising PSA levels, particularly focussing on overall survival.

**Methods:**

Two hundred ninety-five patients with localized prostate cancer were either treated with low-dose-rate (LDR) brachytherapy with I-125 seeds as monotherapy (*n* = 94; 145Gy), high-dose-rate (HDR) brachytherapy with Ir-192 as a boost to external beam RT (*n* = 66; 50.4Gy in 1.8Gy fractions EBRT + 18Gy in 9Gy fractions HDR) or EBRT alone (70.2Gy in 1.8Gy fractions; *n* = 135). “PSA bounce” was defined as an increase of at least 0.2 ng/ml followed by spontaneous return to pre-bounce level or lower, biochemical failure was defined according to the Phoenix definition.

**Results:**

Median follow-up after the end of radiotherapy was 108 months. A PSA bounce showed to be a significant factor for biochemical control (BC) and overall survival (OS) after ten years (BC10 of 83% with bounce vs. 34% without, *p* < 0.01; OS10 of 82% with bounce vs. 59% without bounce, *p* < 0.01). The occurrence of a bounce, a high nadir and the therapy modality (LDR-BT vs. EBRT and HDR-BT + EBRT vs. EBRT) proved to be independent factors for PSA recurrence in multivariate Cox regression analysis. A bounce was detected significantly earlier than a PSA recurrence (median 20 months vs. 32 months after RT; *p* < 0.01; median PSA doubling time 5.5 vs. 5.0 months, not significant). PSA doubling time was prognostically significant in case of PSA recurrence (OS10 of 72% vs. 36% with PSA doubling time ˃ 5 months vs. ≤ 5 months; *p* < 0.01).

**Conclusions:**

Rising PSA levels within the first two years can usually be classified as a benign PSA bounce, with favourable recurrence-free and overall survival rates. PSA doubling time is an important predictor for overall survival following the diagnosis of a recurrence.

## Background

Prostate-specific antigen (PSA) is important for early detection and follow-up of prostate cancer. PSA kinetics after radiotherapy, however, are sometimes difficult to interpret. Especially a rise in PSA upsets patients and their physicians and can lead to an unnecessary secondary therapy.

Several definitions have been established to distinguish a temporary rise in PSA from biochemical failure (BF). The American Society for Therapeutic Radiology and Oncology (ASTRO) coined a definition according to which three consecutive increases of PSA after a nadir can be interpreted as a biochemical failure [1]. This definition was criticized as the occurrence of BF was back-dated [2]. Moreover, the definition depends on the duration of follow-up [3] and cannot be reliably applied to patients with a hormonal therapy (HT) [4].

An increase in an assigned value above a nadir is a more reliable definition [5]. ASTRO therefore provided a second definition of biochemical failure called “Phoenix”, which is based on a rise by 2 ng/ml or more above the nadir and includes patients with an additional hormonal therapy (AHT) [6].

Comparisons of external beam radiotherapy (EBRT) with brachytherapy (BT) have compared biochemical failure rates. The majority of studies did not reveal a significant difference between EBRT and BT [7–9]. Several studies have demonstrated an advantage for BT [10, 11] and only a single study showed better results for EBRT [12]. All prospective randomized studies resulted in improved biochemical failure free survival for BT [13–15].

Increasing PSA levels during follow-up do not always result in a biochemical failure. The onset of steadily rising PSA may be difficult to distinguish from an isolated fluctuation, or “bounce”. PSA levels may rise after the release of antiandrogen hormonal therapy (HT) [4].

A bounce is defined as a brief rise in PSA with a subsequent decline [16]. A bounce has often been reported for patients after low-dose-rate brachytherapy (LDR-BT) and seems to be a favourable predictive factor for BF free survival [17, 18]. Consequences for overall survival rates are not known.

Overall survival is one of the most important clinical endpoints that is not considered in many prostate cancer studies. This endpoint requires long follow-up periods in a patient population treated with curative intent. In contrast to disease specific survival, the definition of overall survival can never be subjective or dependent on a specific definition.

The aim of this study was to evaluate the long-term prognostic significance of rising PSA levels during the follow-up after EBRT or BT, particularly focussing on overall survival.

## Methods

### Patients

This study was based on 295 patients with cT1-3N0M0 prostatic carcinoma (Table 1), who were either treated in the years 2000–2003 with EBRT, a combination of high-dose-rate brachytherapy (HDR-BT) with EBRT or a low-dose-rate brachytherapy (LDR-BT) only.

**Table 1:** Baseline patient characteristics

	All (*n* = 295)	LDR-BT (*n* = 94)	HDR-BT + EBRT (*n* = 66)	EBRT (*n* = 135)
Patient age median (range)	71(49–83)	69(49–81)	72(63–81)	71(52–83)
Follow-up period/months median (range)	108(7–157)	120(8–150)	111(7–157)	105(9–149)
T stage > 2a	21%	5%	36%	25%
Gleason score >6	13%	3%	18%	17%
Primary PSA/ng/ml median (range)	9(1–300)	7(1–15)	13(1–300)	10(1–150)
Low risk patients^a^	43%	65%	27%	35%
Intermediate risk patients^b^	29%	35%	24%	26%
High risk patients^c^	29%	0%	49%	39%
NHT	44%	35%	56%	47%
NHT/months median (range)	4(1–28)	3(1–8)	5(1–28)	5(1–18)

^a^No risk factors: PSA < 10 ng/ml, Gleason score < 7, cT-stage <2b

^b^One risk factors: PSA 10–20 ng/ml or Gleason score = 7 or cT-stage = 2b/c

^c^Two risk factors or PSA > 20 ng/ml or Gleason score >7 or cT-stage >2b/c

The specific treatment was selected after consultation of the patient with the referring urologist and finally the radiation oncologist and the urologist in our centre. Patients referred for LDR-BT usually came long distances to our centre.

Patients were classified according risk factors: Patients without risk factors (PSA < 10 ng/ml, Gleason score <7, cT stage <2b) were in the low risk group, patients with one risk factor (PSA 10–20 ng/ml or Gleason score =7 or cT-stage =2b/c) in the intermediate risk group and patients with two risk factors or PSA > 20 ng/ml or Gleason-Score > 7 or cT >2b/c in the high risk group. High risk patients were always excluded from LDR-BT as monotherapy. All high risk patients received a bone scan and a computed tomography of the abdomen for staging.

### Treatment

The referring urologist usually decided about the indication for neoadjuvant hormonal therapy (NHT). In case of a NHT different agents have been used: luteinizing hormone-releasing hormone (LHRH) agonist (3-monthly depot preparations) in 58% of cases, antiandrogens in 13% of cases and an orchiectomy in 9% of cases. Only in 26 cases (9% of the total patient population) hormonal therapy was continued after RT. The prescription dose to the prostate for permanent LDR-BT was 145 Gy. A median number of 54 sources with a median activity of 0.64 mCi has been implanted in a modified peripheral loading technique.

An Ir-192 stepping source from an afterloader with a nominal activity of 370 GBq was used for temporary HDR-BT. All patients received two fractions to deliver a total minimum dose of 18 Gy to the prostate with 7 days between each fraction. EBRT started within three weeks after brachytherapy. Three dimensional treatment plans were calculated using a four-field box technique wit 15 MeV photons and a multi-leaf collimator. The planning target volume (PTV) was required to be enclosed by the 90% isodose relative to the ICRU (International Commission on Radiation Units and Measurements) reference point with a margin of 1.5 cm in the anterior/lateral and 1 cm in the craniocaudal and dorsal directions to the clinical target volume (CTV = prostate and seminal vesicles). Image guidance techniques for prostate localization have not been used. The total median dose to the prostate in the reference point was 50.4 Gy at 1.8 Gy daily fractions. For EBRT without additional brachytherapy, the same technique was used up to a median dose of 70.2 Gy at 1.8 Gy fractions.

### Evaluation of PSA kinetics

All patients had a pre-treatment PSA measurement. PSA-values and information about a local recurrence, metastases or additional treatment after radiotherapy were collected in regular intervals, usually from the patient (mail or personal interview) or his urologist, sometimes general practitioner.

Changes of PSA values were analyzed according to the following definitions:

1. Nadir: the lowest PSA value measured after the end of the radiotherapy, excluding values after initiation of a salvage hormonal treatment.
2. Biochemical failure (BF): a rise by 2 ng/ml or more above the nadir (Phoenix definition) [6].
3. Bounce: a PSA rise ≥ 0.2 ng/ml followed by spontaneous return to pre-bounce level or lower [17].

### Statistical analysis

Statistical analysis was performed using IBM SPPS Statistics 22.0 software.

PSA doubling time (PSADT) was calculated by using the last PSA value before a PSA rise and the following PSA value according to the following formula: PSADT = [LN(2) x (date 1 – date 2)] / [LN (PSA2) – LN (PSA1)] [19].

Differences between PSA levels or doubling times in subgroups were evaluated using a *t*-test. A forward multivariate logistic regression analysis was used to evaluate the impact of factors for reaching a specific PSA nadir. Kaplan-Meier analysis was used to determine the influence of specific events (nadir, bounce, biochemical failure) on survival. Comparison between subgroups was made with the log-rank test. Different factors were tested for their impact on survival rates in a multivariate Cox regression analysis.

All p-values reported are two-sided; *p* < 0.05 is considered significant.

## Results

The 10-year biochemical recurrence free, metastasis free, disease specific (percentage in the study group who have not died from prostate cancer) and overall survival rates in the total patient population were 53, 89, 90 and 66%, respectively.

### Nadir

Patients with LDR-BT reached a mean nadir of 0.05 ng/ml and patients with HDR-BT + EBRT a mean nadir of 0.1 ng/ml. This is significantly lower in comparison to patients treated with EBRT alone, with a mean nadir of 0.52 ng/ml (*p* < 0.01). The nadir was reached averagely 32 months after LDR-BT, 31 months after HDR-BT + EBRT and 19 months after EBRT. The last nadir for a patient of the HDR-BT + EBRT group occurred after 86 months.

After ten years of follow-up patients with a nadir < 0.17 ng/ml (median nadir of all patients) had significantly higher biochemical recurrence free survival (BC10) than patients with a higher nadir (LDR-BT: 92% vs. 22%, *p* < 0.01; HDR-BT + EBRT: 80% vs. 27%, *p* < 0.01; EBRT: 64% vs. 18%, *p* < 0.01). A multivariate analysis showed LDR-BT vs. EBRT and AHT to be independent factors to reach a nadir of less than 0.17 ng/ml (Table 2). PSA nadir was a significant predictor for disease specific and overall survival (DSS10 of 98% vs. 83%, *p* < 0.01, and OS10 of 78% vs. 55% with a nadir of <0.17 ng/ml vs. ≥0.17 ng/ml, *p* < 0.01).

**Table 2:** Significant factors for PSA nadir <0.17 ng/ml in multivariate analysis

	Factor	Hazard ratio	95% Confidence interval	*p*-value
Nadir <0.17 ng/ml	AHT	2.6	1.5–4.4	*p* < 0.01
	Treatment			
	LDR-BT vs. EBRT	6.9	3.8–12.9	*p* < 0.01
	HDR-BT + EBRT vs. EBRT	3.4	1.8–6.4	*p* < 0.01

### Bounce

**Fig. 1:**
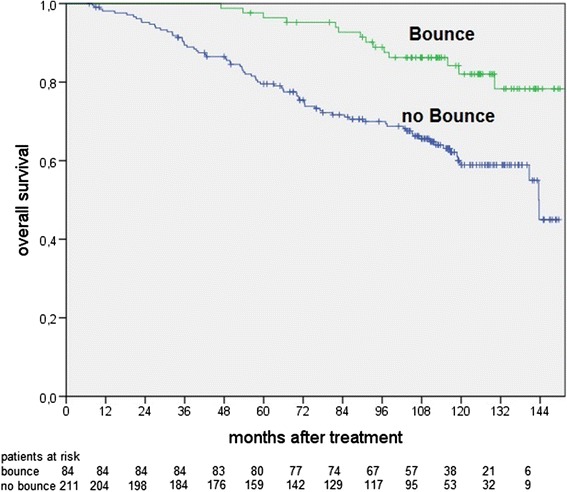
Overall survival for patients with or without a PSA bounce (*p* < 0.01)

A PSA bounce proved to be a significant factor for biochemical control and overall survival after ten years. Patients with a bounce showed better results for recurrence free, disease specific and overall survival (BC of 83% vs. 34%, *p* < 0.01, DSS10 of 99% vs. 86%, *p* < 0.01, OS10 of 82% with bounce vs. 59% without bounce, *p* < 0.01, Fig. 1). A similar prognostic benefit resulted if patients without hormonal therapy were excluded (OS10 pf 83% vs. 62%; *p* = 0.04).

The LDR-BT group had significantly more bounces than the other groups during the follow-up. PSA bounces occured only during the first six years (bounce occurrence after six years with LDR-BT 42% vs. with HDR-BT + EBRT 24% *p* = 0.03, vs. with EBRT 25% *p* < 0.01). Focusing only on the respective treatments, a PSA bounce showed to have a statistically significant effect on overall survival for patients treated with HDR-BT + EBRT (OS10 of 43% vs. 84%; *p* < 0.01) and EBRT (OS10 of 58% vs. 83%; *p* = 0.02), but not patients treated with LDR-BT (OS10 of 76% vs. 82%; *p* = 0.36).

An analysis of patients with a bounce (without BF) and patients with BF (independently from the occurrence of a bounce) revealed a significant difference in PSA kinetics: a bounce was detected significantly earlier than a PSA recurrence (median: 20 months vs. 32 months after RT; *p* < 0.01). The PSA doubling time was comparable: median 5.0 vs. 5.5 months in case of a recurrence vs. a bounce (difference not significant).

### Biochemical failure

Biochemical failure free survival rates are significantly different between treatment groups in the long-term follow-up. 71% of the patients with LDR-BT remained recurrence-free 10 years after treatment, in contrast to HDR-BT + EBRT with 58% (*p* = 0.04) and EBRT with 33% (*p* < 0.01). However, the results were dependent on the prognostic risk groups. No significant differences between the therapy groups were found for the diagnosis of metastases during follow-up (10-year metastasis free survival: LDR-BT 94%, HDR-BT + EBRT 90%, EBRT 83%; *p* = 0.05 for comparison of LDR-BT vs. EBRT).

**Fig. 2:**
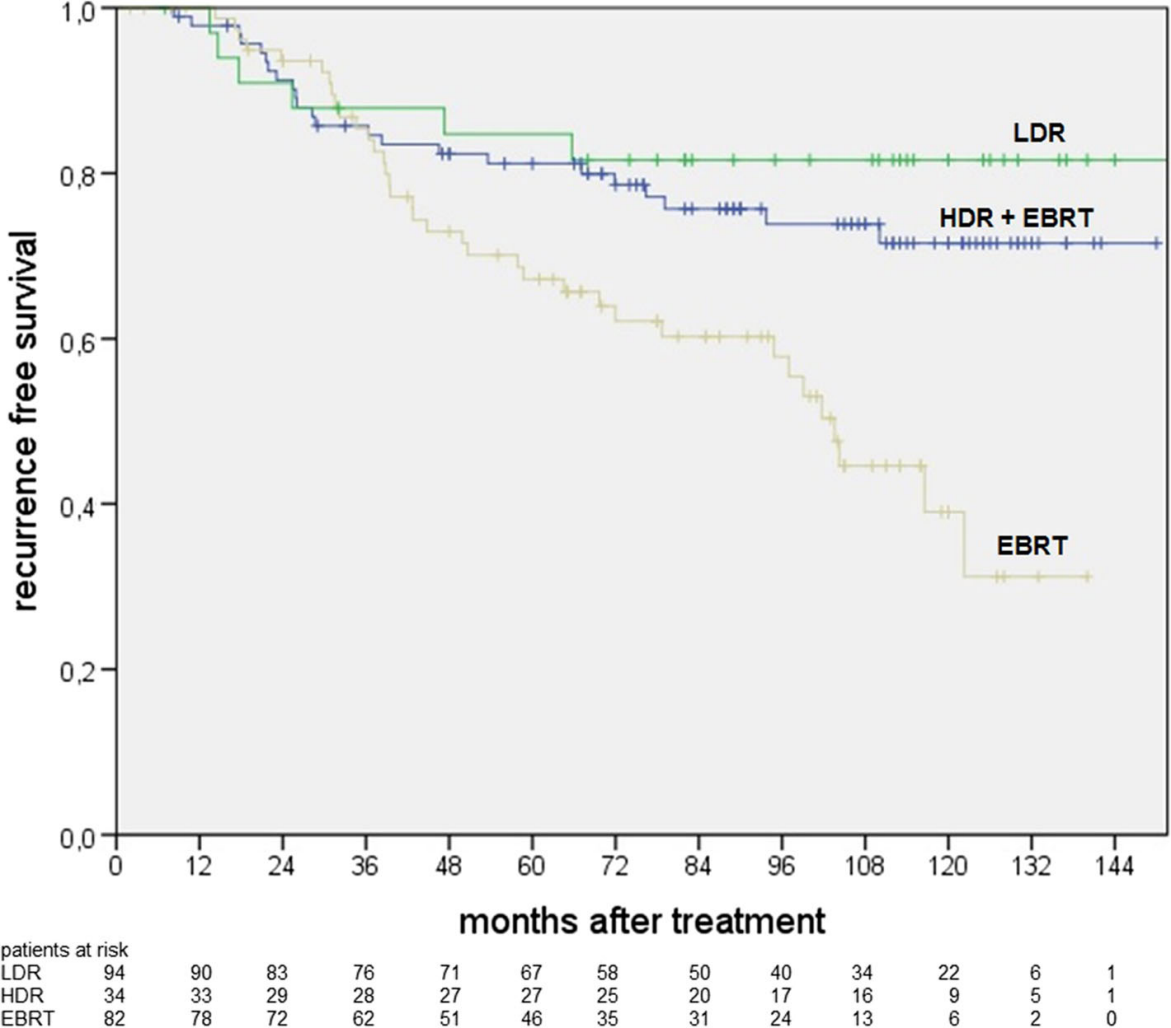
Biochemical recurrence free survival for low and intermediate risk patients (significantly higher rates for LDR vs. EBRT, *p* = 0.03, and HDR-BT vs. EBRT, *p* = 0.03)

While patients after HDR-BT + EBRT showed the best result concerning freedom from biochemical recurrence in the low-risk-group (HDR-BT + EBRT 94% free from biochemical failure vs. LDR-BT 68%; vs. EBRT 67%, *p* < 0.01), LDR-BT was the best in the intermediate group (78% vs. HDR-BT + EBRT 67%; vs. EBRT 33%, *p* < 0.01). Figure 2 summarizes the biochemical recurrence free survival for low and intermediate risk patients, depending on treatment technique. There was no statistically significant difference in the high risk group between HDR-BT and EBRT.

A multivariate Cox regression analysis revealed the occurrence of a bounce, a low nadir value, LDR-BT vs. EBRT and HDR-BT + EBRT vs. EBRT to be independent protective factors against biochemical failure.

The 10-year overall survival (OS10) with or without a biochemical recurrence was 74 and 54%, respectively (*p* < 0.01). The PSA doubling time proved to have an impact on overall survival after biochemical failure. The classification of all patients according to the median doubling time of 5 months showed a better disease specific and overall survival for patients with a longer doubling time (DSS10 of 87% vs. 68%, *p* = 0.01, OS10 of 72% vs. 36%, *p* < 0.01, with a PSA doubling time > 5 months vs. ≤ 5 months).

## Discussion

Knowledge about PSA kinetics after radiotherapy for localized prostate cancer is very important for the assessment of the therapy success, tumour control and detection of recurrences.

Following treatments, patients usually reach a nadir PSA [19]. The nadir values after a follow-up were similar to the values of a prior analysis [11] as a nadir is usually reached within the first three years after radiotherapy: with LDR-BT averagely after 32 months, with HDR-BT + EBRT averagely after 31 months and with EBRT averagely after 19 months. A nadir after EBRT is significantly higher and it is reached sooner in comparison to brachytherapy. Because of a higher biologically effective dose (BED) with brachytherapy, both malignant and benign prostate cells are destroyed. Owing to longer cell cycle of benign cells, cell death takes longer. These facts explain the lower nadir and the longer time to reach a nadir for patients after brachytherapy [11].

A low nadir is, as also shown in other studies, a favourable prognostic factor for recurrence-free survival [11, 19]. Multivariate analysis has shown AHT and LDR-BT vs. EBRT to be independent factors for the occurrence of a nadir ≤ 0.17 ng/ml. PSA nadir also predicted overall survival in this study.

Current guidelines recommend AHT for 2–3 years for high risk patients treated with EBRT. A subset of intermediate risk patients might benefit from a short term (4–6 months) hormonal therapy [20].

Rising PSA values alarm patients and treating physicians. A differentiation between bounce and biochemical failure can be difficult. A temporary PSA rise can occur after neoadjuvant hormonal therapy [19], usually in the two years of follow up. As 44% of patients received a neoadjuvant hormonal therapy, this factor has a major impact in this analysis. Nevertheless, the prognostic effect of a PSA bounce has also been found excluding these patients.

A bounce, defined as a rise of >0.2 ng/ml with a subsequent decline, can be idiopathic or induced by increasing testosterone levels, caused by proctitis or instrumentation [19, 21]. These factors, together with reactive changes within the prostate after high dose treatment, occur especially within the first 2–3 years, as also shown in this study.

A bounce is a prognostically favourable factor for biochemical control. This effect was also demonstrated in other studies, especially after LDR-BT [11, 22]. Biochemical failure free survival was higher after LDR-BT in comparison to the other two therapies in this study. However, LDR-BT was predominantly used for low risk patients with a favourable prognosis. Most probably as a result of a favourable prognosis, a bounce reached the level of statistical significance for overall survival only for the other two treatment technique groups.

The comparison of patients with a bounce and patients with BF revealed significant differences for PSA kinetics. While a bounce is detected averagely after 21 months, a biochemical recurrence occurred averagely after 32 months. No difference in PSA doubling time in case of a bounce, as compared to a recurrence, was detected. Similar results were found in another study, reporting a bounce 15 months after treatment and biochemical failure, also defined using the Phoenix definition, after 28 months [18]. Another study reported a comparable mean time of 30 months for the occurrence of biochemical recurrence after HDR-BT + EBRT [23]. These findings indicate that increasing PSA levels within the first two years of follow-up are most probably benign PSA rises.

Biochemical failure occurred less frequently after LDR-BT in comparison to HDR-BT + EBRT or EBRT. The analysis of individual risk groups showed a significant advantage for HDR-BT + EBRT over EBRT in the low-risk group and for LDR-BT over EBRT in the intermediate-risk group. The benefit of a dose escalation, as reached with brachytherapy, is well known. A selection bias might exist in this study in the individual risk groups, as for example patients qualify for LDR-BT only with a specific maximum prostate volume. Nevertheless, favourable outcomes after LDR-BT [24, 25] and an advantage over EBRT [10, 11, 26] have been reported in the literature, including a recently published randomized study [13]. Multivariate analysis revealed the occurrence of a bounce, a low nadir value, LDR-BT vs. EBRT and HDR-BT + EBRT vs. EBRT to be independent factors regarding biochemical failure.

The PSA doubling time in case of a recurrence is crucial for the assessment of patient prognosis. Patients with a PSA doubling time > 5 months showed a 10-years-survival rate of 72%, in contrast to a survival rate of 36% for patients with a PSA doubling of less than 5 months. A prognostic impact of the PSA doubling time was also reported in another study, demonstrating a 10-year disease-specific survival rate of 30% for patients with a PSA failure and a PSA doubling time ≤6 months vs. 67% and 98% for those with PSA failure and doubling time >6-10 months and >10 months [27].

## Conclusions

Biochemical recurrence free survival rates are dependent on the treatment dose and technique. PSA nadir is reached mostly within the first three years during follow-up. A PSA rise within the first two years can usually be classified as a benign and favourable PSA bounce, associated with significantly better overall survival in comparison to patients without a bounce. PSA doubling time is an important predictor for overall survival following the diagnosis of a recurrence.

The results of this study are important for the decision about further diagnostics or additional treatments during follow-up after primary radiotherapy.

## Abbreviations

AHT: Adjuvant/Additional hormonal therapy
ASTRO: American Society for Therapeutic Radiology and Oncology
BC: Biochemical control
BED: Biologically effective dose
BF: Biochemical failure
BT: Brachytherapy
CTV: Clinical target volume
DSS: Disease specific survival
EBRT: External beam radiotherapy
HDR-BT: High-dose-rate brachytherapy
HT: Hormonal therapy
ICRU: International Commission on Radiation Units and Measurements
LDR-BT: Low-dose-rate brachytherapy
LHRH: Luteinizing hormone-releasing hormone
NHT: Neoadjuvant hormonal therapy
OS: Overall survival
PSA: Prostate-specific antigen
PTV: Planning target volume
RT: Radiotherapy

## References

[1] ASTRO Consensus Panel (1997). Consensus statement: guidelines for PSA following radiation therapy. American Society for Therapeutic Radiology and Oncology Consensus Panel. Int J Radiat Oncol Biol Phys.

[2] Horwitz EM, Uzzo RG, Hanlon AL, Greenberg RE, Hanks GE, Pollack A (2003). Modifying the American Society for Therapeutic Radiology and Oncology definition of biochemical failure to minimize the influence of backdating in patients with prostate cancer treated with 3-dimensional conformal radiation therapy alone. J Urol.

[3] Vicini FA, Kestin LL, Martinez AA (1999). The importance of adequate follow-up in defining treatment success after external beam irradiation for prostate cancer. Int J Radiat Oncol Biol Phys.

[4] Buyyounouski MK, Hanlon AL, Eisenberg DF, Horwitz EM, Feigenberg SJ, Uzzo RG, Pollack A (2005). Defining biochemical failure after radiotherapy with and without androgen deprivation for prostate cancer. Int J Radiat Oncol Biol Phys.

[5] Fitch DL, McGrath S, Martinez AA, Vicini FA, Kestin LL (2006). Unification of a common biochemical failure definition for prostate cancer treated with brachytherapy or external beam radiotherapy with or without androgen deprivation. Int J Radiat Oncol Biol Phys.

[6] Roach M, Hanks G, Thames H, Schellhammer P, Shipley WU, Sokol GH, Sandler H (2006). Defining biochemical failure following radiotherapy with or without hormonal therapy in men with clinically localized prostate cancer: recommendations of the RTOG-ASTRO Phoenix Consensus Conference. Int J Radiat Oncol Biol Phys.

[7] Burdick MJ, Reddy CA, Ulchaker J, Angermeier K, Altman A, Chehade N, Mahadevan A, Kupelian PA, Klein EA, Ciezki JP (2009). Comparison of biochemical relapse-free survival between primary Gleason score 3 and primary Gleason score 4 for biopsy Gleason score 7 prostate cancer. Int J Radiat Oncol Biol Phys.

[8] Pe ML, Trabulsi EJ, Kedika R, Pequignot E, Dicker AP, Gomella LG, Valicenti RK (2009). Effect of percentage of positive prostate biopsy cores on biochemical outcome in low-risk PCa treated with brachytherapy or 3D-CRT. Urology.

[9] Vassil AD, Murphy ES, Reddy CA, Angermeier KW, Altman A, Chehade N, Ulchaker J, Klein EA, Ciezki JP (2010). Five year biochemical recurrence free survival for intermediate risk prostate cancer after radical prostatectomy, external beam radiation therapy or permanent seed implantation. Urology.

[10] Pickles T, Keyes M, Morris WJ (2010). Brachytherapy or conformal external radiotherapy for prostate cancer: a single-institution matched-pair analysis. Int J Radiat Oncol Biol Phys.

[11] Pinkawa M, Piroth MD, Holy R, Fischedick K, Schaar S, Borchers H, Heidenreich A, Eble MJ (2010). Prostate-specific antigen kinetics following external-beam radiotherapy and temporary (Ir-192) or permanent (I-125) brachytherapy for prostate cancer. Radiother Oncol.

[12] Eade TN, Horwitz EM, Ruth K, Buyyounouski MK, D’Ambrosio DJ, Feigenberg SJ, Chen DY, Pollack A (2008). A comparison of acute and chronic toxicity for men with low-risk prostate cancer treated with intensity-modulated radiation therapy or (125)I permanent implant. Int J Radiat Oncol Biol Phys.

[13] Sathya JR, Davis IR, Julian JA, Guo Q, Daya D, Dayes IS, Lukka HR, Levine M (2005). Randomized trial comparing iridium implant plus external-beam radiation therapy with external-beam radiation therapy alone in node-negative locally advanced cancer of the prostate. J Clin Oncol.

[14] Hoskin PJ, Rojas AM, Bownes PJ, Lowe GJ, Ostler PJ, Bryant L (2012). Randomised trial of external beam radiotherapy alone or combined with high-dose-rate brachytherapy boost for localised prostate cancer. Radiother Oncol.

[15] Morris WJ, Tyldesley S, Rodda S, Halperin R, Pai H, McKenzie M, Duncan G, Morton G, Murray N, Hamm J, Murray N (2017). ASCENDE-RT*: a multicenter, randomized trial of dose-escalated external beam boost for high- and intermediate-risk prostate cancer. Int J Radiat Oncol Phys.

[16] Akyol F, Ozyigit G, Selek U, Karabulut E (2005). PSA bouncing after short term androgen deprivation and 3D-conformal radiotherapy for localized prostate adenocarcinoma and the relationship with the kinetics of testosterone. Eur Urol.

[17] Crook J, Gillan C, Yeung I, Austen L, McLean M, Lockwood G (2007). PSA kinetics and PSA bounce following permanent seed prostate brachytherapy. Int J Radiat Oncol Biol Phys.

[18] Mitchell DM, Swindell R, Elliott T, Wylie JP, Taylor CM, Logue JP (2008). Analysis of prostate-specific antigen bounce after I(125) permanent seed implant for localised prostate cancer. Radiother Oncol.

[19] Pinkawa M, Fischedick K, Piroth MD, Gagel B, Borchers H, Jakse G, Eble MJ (2007). Prostate-specific antigen kinetics after brachytherapy or external beam radiotherapy and neoadjuvant hormonal therapy. Urology.

[20] Heidenreich A, Bastian PJ, Bellmunt J, Bolla M, Joniau S, van der Kwast T, Mason M, Matveev V, Wiegel T, Zattoni F, Mottet N (2013). EAU guidelines on prostate acner. Part 1: screening, diagnosis, and local treatment with curative intent-update 2013. Eur Urol.

[21] Das P, Chen MH, Valentine K, Lopes L, Cormack RA, Renshaw AA, Tempany CM, Kumar S, D’Amico AV (2002). Using the magnitude of PSA bounce after MRI-guided prostate brachytherapy to distinguish recurrence, benign precipitating factors, and idiopathic bounce. Int J Radiat Oncol Biol Phys.

[22] Patel C, Elshaikh MA, Angermeier K, Ulchaker J, Klein EA, Chehade N, Wilkinson DA, Reddy CA, Ciezki JP (2004). PSA bounce predicts early success in patients with permanent iodine-125 prostate implant. Urology.

[23] Prada PJ, Gonzalez H, Fernandez J, Jimenez I, Iglesias A, Romo I (2012). Biochemical outcome after high-dose-rate intensity modulated brachytherapy with external beam radiotherapy: 12 years of experience. BJU Int.

[24] Zuber S, Weiss S, Baaske D, Schope M, Stevens S, Bodis S, Zwahlen DR (2015). Iodine-125 seed brachytherapy for early stage prostate cancer: a single-institution review. Radiat Oncol.

[25] Voulgaris S, Nobes JP, Laing RW, Langley SE (2008). State-of-the-art: prostate LDR brachytherapy. Prostate Cancer Prostatic Dis.

[26] Ferrer M, Guedea F, Suarez JF, de Paula B, Macias V, Marino A, Hervas A, Herruzo I, Ortiz MJ, de LJ P, Sancho G, Boladeras A, Ayala A, Craven-Bratle J, Avila M, Cunillera O, Pardo Y, Alonso J, Aguilo F (2013). Quality of life impact of treatments for localized prostate cancer: cohort study with a 5 year follow-up. Radiother Oncol.

[27] Stock RG, Cesaretti JA, Stone NN (2006). Disease-specific survival following the brachytherapy management of prostate cancer. Int J Radiat Oncol Biol Phys.

